# Twenty Years After Glioblastoma Multiforme Diagnosis: A Case of Long-Term Survival

**DOI:** 10.7759/cureus.16061

**Published:** 2021-06-30

**Authors:** Omar Rabab'h, Ali Al-Ramadan, Jawad Shah, Hugo Lopez-Negrete, Abeer Gharaibeh

**Affiliations:** 1 Research, Insight Research Institute, Flint, USA; 2 Research, University of Michigan-Flint, Center for Cognition and Neuroethics, Flint, USA; 3 Neurology, Insight Research Institute, Flint, USA; 4 Neurology, University of Michigan-Flint, Center for Cognition and Neuroethics, Flint, USA; 5 Neurosurgery, Insight Research Institute, Insight Institute of Neurosurgery & Neuroscience, Flint, USA; 6 Neurosurgery, University of Michigan-Flint, Center for Cognition and Neuroethics, Flint, USA; 7 Neurosurgery, Michigan State University, East Lansing, USA; 8 Neurosurgery, Hurley Medical Center, Flint, USA

**Keywords:** glioblastoma multiforme, varicella zoster virus, immune surveillance, long term survival, oncolytic viruses

## Abstract

Glioblastoma multiforme (GBM) is an aggressive tumor that has a poor prognosis with a median survival of 15 months with treatment and 3-4 months without treatment. Subsets of patients are found to survive longer than two years, some survivors lived more than 10 years, and rare cases survived 20 years or more with treatment. Better prognosis has been found to be associated with many factors. Some of these factors are related to patients' characteristics, biological factors that impact tumor aggressiveness, and/or factors associated with treatment. However, the exact contribution for extended survival is still not known. Finding the factors that have a strong impact on the long survival is of high importance and can help give hope to better treat glioblastoma cases. In this report, we present a case of a glioblastoma patient who was diagnosed at the age of 47 years with more than 20-year survival. We further discuss the suggested factors that may have contributed to a better prognosis with a focus on the possible role of varicella-zoster infection in mediating long-term survival.

## Introduction

Glioblastoma multiforme (GBM) is considered the most aggressive and most common primary brain tumor in adults. It constitutes 14.6% of all brain tumors and 48.3% of malignant brain tumors [1]. According to the World Health Organization classification, glial tumors are classified into four grades (I-VI) depending on their characteristics [2]. GBM is considered a grade IV glioma and accounts for the majority of glioma cases (57.3%) [1,2]. GBM cases are diagnosed based on imaging and histological findings of the tumor [3]. The tumor originates in cerebral hemispheres in 95% of the cases [4], and it can involve the corpus callosum bilaterally which gives the famous butterfly appearance of GBM on imaging [5]. Management of GBM patients includes surgical resection of the tumor, chemotherapy, and radiotherapy [3]. Although some studies showed a trend toward improving the survival rate of GBM patients over time, GBM has a poor prognosis [6-8]. Despite the improvement in short-term survival rate, the five-year survival rate remained around 6.8% of the cases which is considered the lowest among brain tumors [1,8]. Long-term survival with 10 years and more accounts for less than 1% of cases [9]. Age represents the most important factor for long survival with most of the reported cases being for patients younger than 40 years of age [9]. Novel therapeutics are being investigated for GBM treatment and some show promising results [8]. The median survival and two-year relative survival rate improved from 12 months and 15% for those diagnosed in 2000-2001 to 15 months and 26% for those diagnosed in the 2005-2006 period [6,7]. The use of carmustine wafers, bevacizumab, and temozolomide, and advances in surgical and radiation techniques are thought to be major contributors to improved short-term survival [6,7]. Immunotherapy using immune checkpoint inhibitors, tumor vaccines and oncolytic viruses are among those being tested and they hold promising results [8].

In this case report, we present a rare case of a patient who was diagnosed with GBM 20 years ago and survived without tumor recurrence.

## Case presentation

In September 1999, a previously healthy 47-year-old Caucasian male patient presented to the outpatient clinic with a bilateral temporal headache and feeling of pressure around his eyes. The patient was diagnosed with a tension headache at that time and was prescribed analgesics. Few weeks later, he started to develop changes in smell perception in addition to headaches. These symptoms progressed for two months during which the patient did not seek medical attention. In mid-January 2000, the patient had an episode of syncope and was brought to the emergency department. The patient underwent a brain CT scan and was found to have increased intracranial pressure and a lesion on the right side. Brain magnetic resonance imaging (MRI) was performed and revealed a contrast-enhancing lesion in the right parieto-occipital region. The patient underwent craniotomy, and the lesion was resected without complications. Histopathological examination of the resected tumor revealed grade IV glioma (GBM) which was confirmed at multiple pathology laboratories. After that, the patient received external beam radiotherapy treatment given in 30 fractions with 2 Gy per fraction over six weeks and chemotherapy (temozolomide) for 26 months. Later, in 2001, the patient noticed a small skin lesion in his right arm which was found to be melanoma. The lesion was removed without any complications. In 2002, the patient developed a seizure, and was transported to the hospital. Imaging showed that the patient had a scar/necrotic tissue in the previously resected GBM tumor area which was then removed. 

**Figure 1:**
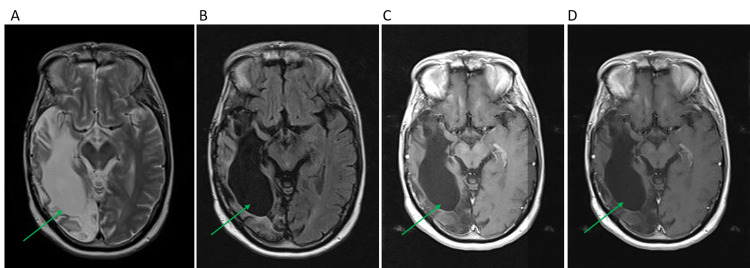
Pre- and Post-Contrast Brain MRI Brain MRI was done for the patient in a follow-up visit after 20 years of the GBM tumor resection. Encephalomalacia is seen in the right frontotemporal lobe. No evidence of glioblastoma recurrence. A: axial flair T1, B: axial T2, C and D: post-contrast axial T1.

Three years ago, in 2018, the patient had sudden weakness in his left leg and seizure. Imaging findings were consistent with chronic subdural hematoma with no evidence of tumor recurrence. The most recent follow up of the patient after 20 years of tumor removal re-affirmed that the patient was generally in a good status and free of the tumor. He was oriented to place, time and person, and his neurological examination showed some degree of psychomotor slowing, left-sided homonymous hemianopia, and difficulty in hearing. The patient’s words while speaking were comprehended and appropriate, but he has a short-term memory deficit and some difficulty with executive functioning. The patient reported a blank-out seizure once a year although he was on anti-epilepsy medications. Other neurological investigations including cranial nerves, muscle strength, and gait were normal. Brain MRI showed encephalomalacia, mild cortical volume loss, and stable chronic microvascular ischemic changes (Figure 1). MRI did not show any evidence of tumor recurrence. 

The patient is 67 years old now, 20 years after the initial diagnosis of GBM which is considered a rare long survival in GBM cases. Thus, the case was further analyzed for more details that could reveal factors that may be associated with such long survival. The patient's past medical history showed that he had a history of chickenpox infection and shingles when he was in his early thirties which was before developing his initial symptoms.

## Discussion

GBM carries a poor prognosis even with the introduction of post-surgical radiotherapy and chemotherapy treatments. The median relative survival among GBM patients is 15 months and the two-year relative survival is 25% [6]. Only 0.71% of patients survive more than 10 years, [9] and 20 years of survival, as in this case, is rare and was reported in only a few cases [9-12].

The suggested factors that are associated with better prognosis are younger age at diagnosis, higher Karnofsky Performance Scale (KPS), complete surgical resection, smaller tumor size, and radiotherapy initiation within six weeks of surgery [13,14]. There is an inverse association between at what age the GBM was diagnosed and prognosis. The 10-year relative survival rate decreased from 15.2% for patients between the ages of 20-44 years to 5.7% for patients between the ages of 45-54 years, respectively [1]. Molecular features of the tumor such as 6-methylguanine-DNA methyltransferase (MGMT) promoter methylation and isocitrate dehydrogenase 1/2 (IDH1/2) mutations are also associated with better prognosis [2,15,16]. Patients with MGMT methylation are associated with better survival and response to temozolomide treatment [15]. The co-existence of IDH and MGMT methylation is associated with a better prognosis and predicts the responsiveness to chemotherapy and surgical resection [16].

Our patient was 47-year-old when he was diagnosed with GBM, which is relatively younger in comparison to the median age of diagnosis which is 59 years [3]. However, most patients with a long survival of more than 10 years were under the age of 40 [9]. The patient was in good health pre- and post-operatively, there were no known neurological deficits at that time and his KPS score was around 80. The tumor also was localized, and complete surgical resection was achieved which was followed by radiotherapy and temozolomide treatment. Molecular factors were not assessed due to the date of diagnosis, and the patient did not consent to the investigational genomic panel at the time of writing this case report.

Interestingly, having chickenpox and shingles infections which are caused by the varicella-zoster virus (VZV) prior to the tumor diagnosis might have also played a role in this case. There is increasing evidence that immune surveillance is a long-known arm of the defense against tumor development and progression, and it has been harnessed for cancer treatment [17]. Boosting anti-tumor immunity by means of concurrent bacterial infection with GBM has been proposed to be a mechanism to mediate long-term survival in GBM patients [18]. Several studies pointed to the inverse association between VZV infection and glioma [19,20]. Studies showed that VZV is associated with 21% lower glioma risk development [19]. This effect is more prominent in younger age patients and high-grade gliomas [19]. A possible explanation is the presence of cross-reactive antigens between VZV and tumor cells [20]. Another explanation is a direct cytolytic effect which can be activated when infected cells undergo malignant transformation [20,21]. An encouraging finding was noted in a phase 1 trial of using oncolytic virus polio-rhinovirus chimera (PVSRIPO) in GBM treatments [22]. Desjardins and colleagues investigated PVSRIPO in 61 patients with recurrent GBM and found that the overall survival at 24 and 36 months was 21% which is higher than historical controls. Notably, some patients remain alive more than 70 months after PVSRIPO infusion [22]. Interestingly, some people also naturally have immunity against shared tumor antigens [23,24]. An example of this is cyclin B1 which is overexpressed in many solid tumors and GBM [25,26]. VZV infection can elicit anti-cyclin B1 immunity, induce cytoplasmic expression of this nuclear protein, and enhance its delivery to dendritic cells through packaging it in virions. This leads to immune cells priming against cyclin B1 [27-31]. Furthermore, studies showed that enhancing anti-cyclin B1 immunity can both prevent and reduce tumor growth in cyclin B1-expressing tumor in mice [24,32]. Our patient was infected with this virus two times prior to the GBM diagnosis which could have played a role in long survival. It is also possible that the immunity was elicited against other shared tumor antigens. However, analysis of cyclin B expression and the presence of anti-cyclin B immune cells or antibodies are needed to support this hypothesis.

Additionally, utilizing viruses that are capable of killing tumor cells is fundamental to the concept of oncolytic viruses. The use of engineered oncolytic viruses to stimulate antitumor immune response has been investigated previously [33]. Several viruses were found to mediate the destruction of GBM tissue through different mechanisms including immunogenic cell death, stimulation of antiviral innate immune response, and adaptive anti-tumor T cell response [33]. Several clinical trials are investigating the potential beneficial effects of oncolytic viruses such as recombinant nonpathogenic PVSRIPO, reovirus, and measles virus [22,33]. VZV has been proposed as a new oncolytic virus candidate for GBM treatment [21].

## Conclusions

Though the extended survival for 20 years in GBM patients is extremely rare, it is encouraging to see cases with long survival as it gives hope for finding a cure for this deadly cancer. We hypothesize that having a history of VZN infection could be a possible contributor to the survival duration in our patient. Although some hidden factors may also play a role, it is tempting to conduct epidemiological studies to analyze the association between VZV infection and its effect on survival in GBM patients. Boosting anti-tumor immune surveillance seems to play a critical role in GBM prognosis and warrants further investigations.
